# Circadian phenotype is associated with diurnal variation in human exercise performance

**DOI:** 10.1007/s00421-026-06352-0

**Published:** 2026-07-15

**Authors:** Akshay Singh, Sanjida Ahmed, Stuart J. Hesketh

**Affiliations:** School of Medicine and Dentistry, University of Lancashire, Victoria Street, Harrington Building, Preston, PR1 2HE UK

**Keywords:** Chronotype, Sleep timing, Exercise timing, Circadian rhythm, Time of day, Performance variability

## Abstract

**Purpose:**

Time-of-day variation in exercise performance is well established, but the contribution of circadian phenotype to the magnitude of these differences remains unclear. This study examined diurnal variation in aerobic exercise performance and determined whether chronotype and sleep timing were associated with morning–evening differences in performance.

**Methods:**

Twenty-four healthy young adults (12 male, 12 female) completed a randomised crossover protocol consisting of a 10-min cycling time trial performed in the morning (08:00–09:00) and evening (17:00–18:00). Pre-trial sleep, diet, and physical activity were standardised, and circadian phenotype was characterised using the Composite Morningness Questionnaire and 7-day actigraphy.

**Results:**

Mean power, peak power, and distance covered were all significantly higher in the evening compared with morning (*p* < 0.01). Chronotype (*r* = − 0.47, *p* = 0.019) and sleep midpoint (*r* = 0.52, *p* = 0.003) were significantly associated with the magnitude of the diurnal performance difference, accounting for 22% and 27% of the variance, respectively. Heart rate, blood lactate, and perceived exertion responses were similar between conditions, indicating comparable physiological strain. Core temperature was higher in the evening (*p* = 0.026), but did not explain performance differences.

**Conclusions:**

These findings demonstrate that endurance exercise performance is enhanced in the evening compared with the morning and suggest that behavioural markers of circadian phenotype are associated with the magnitude of diurnal variation in performance. Time-of-day and circadian phenotype should therefore be considered when optimising exercise testing and prescription.

## Introduction

Time-of-day variation in human exercise performance is well established (Hesketh and Esser 2024), with numerous studies demonstrating superior performance in the late afternoon and early evening compared with the morning across a range of exercise modalities, including endurance (van Moorsel et al. 2016), strength (Douglas et al. 2021), and high-intensity tasks (Chtourou et al. 2011, 2012; Hill et al. 1989; Reilly et al. 2007). These diurnal fluctuations have commonly been attributed to underlying circadian rhythms in physiological and behavioural factors such as core body temperature, neuromuscular function, and perceived exertion (Atkinson and Reilly 1996; Pullinger et al. 2018; Robinson et al. 2013; Souissi et al. 2010). However, despite this general pattern, findings across studies remain variable, with considerable heterogeneity in the magnitude of reported time-of-day effects, and some investigations reporting only small or non-significant differences between morning and evening performance (Bommasamudram et al. 2022; Hesketh et al. 2026; Kang et al. 2023).

A key limitation within the existing literature is the lack of methodological consistency and experimental control. Differences in exercise modality, performance protocols, and the extent to which pre-trial behaviours are standardised make direct comparison between studies challenging and may confound interpretation of time-of-day effects (Bommasamudram et al. 2022). Factors such as prior sleep, habitual physical activity, dietary intake, and caffeine consumption are known to influence exercise performance, yet are not consistently controlled (Tsai et al. 2025; K. C. Vitale et al. 2019). Furthermore, the use of between-subject designs and limited familiarisation procedures in some studies may increase inter-individual variability and obscure true diurnal differences. As such, there remains a need for tightly controlled, within-subject investigations using simple, reproducible protocols to more precisely characterise the magnitude of diurnal variation in human performance.

In addition to methodological considerations, there is increasing interest in the role of circadian phenotype as a potential contributor to time-of-day differences in performance (Facer-Childs and Brandstaetter 2015; K. C. Vitale et al. 2019). Chronotype, reflecting an individual’s preferred timing of sleep and activity, has been proposed as an important determinant of performance capacity across the day (Bommasamudram et al. 2022; J. A. Vitale et al. 2017). Individuals with a later chronotype may demonstrate relatively enhanced performance in the evening, whereas earlier chronotypes may exhibit attenuated or absent diurnal variation (Facer-Childs and Brandstaetter 2015; J. A. Vitale et al. 2017). However, evidence for such effects remains limited, and relatively few studies have combined objective assessment of sleep–wake behaviour with controlled exercise performance testing to directly quantify the contribution of circadian phenotype and the magnitude of diurnal variation in performance under controlled conditions.

Emerging work has also highlighted the importance of circadian timing in the regulation of exercise metabolism and adaptation. For example, Ezagouri et al. (2019) demonstrated that exercise capacity and metabolic responses vary as a function of circadian phase in both animal models and humans, with distinct molecular signatures observed depending on the timing of exercise. While these findings provide important mechanistic insight, translation to controlled human performance studies remains limited, particularly in relation to behavioural proxies of circadian timing and their relationship to functional performance outcomes.

From an applied perspective, understanding whether exercise performance varies meaningfully across the day under controlled conditions, and the extent to which this variation is associated with circadian phenotype, has direct relevance for training prescription, performance testing, and the scheduling of physical activity. Establishing the magnitude of diurnal variation using simple, reproducible exercise protocols, alongside accessible measures of sleep–wake behaviour, may provide a practical framework for individualising exercise timing strategies.

Therefore, the aim of the present study was to examine diurnal variation in aerobic exercise performance and associated physiological responses during a standardised 10-minute cycling time trial in healthy young adults using a randomised, counterbalanced crossover design with tightly controlled pre-trial conditions. A secondary aim was to determine whether inter-individual differences in circadian phenotype, assessed via chronotype questionnaire and actigraphy-derived sleep metrics, were associated with the magnitude of time-of-day differences in performance. It was hypothesised that performance would be enhanced in the evening compared with the morning, and that inter-individual variability in this response would be associated with differences in circadian phenotype.

## Methods

### Participants

Twenty-four healthy young adults (12 males: age 27 ± 4 years, body mass 78 ± 11 kg, height 181 ± 15 cm; and 12 females: age 28 ± 2 years, body mass 65 ± 5 kg, height 170 ± 8 cm) were recruited to participate in this study. Participants were recreationally active individuals, free from known cardiovascular, metabolic, or sleep disorders, and not engaged in structured exercise training programmes. Exclusion criteria included shift work within the previous three months, transmeridian travel within four weeks of testing, and use of medications known to affect sleep or cardiovascular function.

To reduce variability associated with hormonal fluctuations, all female participants were tested between days 10–15 following the onset of menstruation, based on self-report of the first day of their most recent cycle and typical cycle length, which were verified at each laboratory visit. None of the participants were using hormonal contraceptives at the time of the study. Participants were instructed to replicate their dietary intake prior to each experimental trial and to report any deviations from their habitual sleep patterns during the study period. All trials for each participant were conducted at consistent times within the predefined morning (08:00–09:00) and evening (17:00–18:00) testing windows.

All participants provided written informed consent prior to participation. The study was conducted in accordance with the Declaration of Helsinki and received approval from the Ethics Committee of the School of Medicine and Dentistry, University of Lancashire. All procedures complied with institutional standards for the collection, storage, and confidentiality of human data.

### Sample size estimation and justification

A priori sample size estimation was informed by previous studies investigating diurnal variation in exercise performance using within-subject designs, which typically report moderate effect sizes and include sample sizes of approximately 10–20 participants (Chtourou et al. 2012; Souissi et al. 2013, 2020). Based on these data, and assuming a moderate effect size (Cohen’s *d* ≈ 0.6–0.8) for differences in performance between morning and evening conditions, a minimum sample size of 20 participants was estimated to provide > 80% statistical power at an alpha level of 0.05 for the primary outcome. To account for potential dropouts and inter-individual variability in responses, a total of 24 participants were recruited. This sample size is consistent with previous controlled crossover studies examining time-of-day effects on exercise performance and was considered sufficient to detect differences in the primary outcome measure. It should be noted that analyses examining associations between circadian phenotype variables and performance outcomes were exploratory in nature and were not specifically powered. As such, these findings should be interpreted with appropriate caution.

### Study design

A randomised crossover design was employed. Each participant attended the laboratory on three occasions: one familiarisation session followed by two experimental trials performed in the morning (AM; 08:00–09:00) and evening (PM; 17:00–18:00). The order of AM and PM trials was randomised and counterbalanced using a computer-generated randomisation sequence to minimise potential order and learning effects. Trials were separated by a minimum of 72 h to reduce the likelihood of carryover effects. All experimental trials for each participant were conducted at consistent times within the predefined AM and PM testing windows.

Prior to the first experimental trial, participants completed seven consecutive days of actigraphy monitoring using a wrist-worn device (Fitbit Inspire 3, Fitbit Inc., USA), alongside a validated chronotype questionnaire (Composite Morningness Questionnaire, CMQ) (Shahid et al. 2011), to characterise habitual sleep–wake behaviour and circadian preference.

### Pre-trial standardisation

Participants were instructed to maintain their habitual sleep–wake schedule during the monitoring period and between trials. For the 24 h preceding each experimental trial, participants were required to refrain from strenuous physical activity, abstain from alcohol, avoid caffeine consumption for at least 12 h, and replicate their dietary intake and pre-exercise feeding patterns as closely as possible between trials. Participants were instructed to obtain a minimum of 6 h of sleep prior to each trial and to record sleep timing and quality in a diary.

For morning trials, participants arrived at the laboratory within approximately 1–2 h of habitual waking time to minimise variability in the time elapsed since waking. For evening trials, participants were asked to maintain normal daytime activities while avoiding strenuous exercise and caffeine consumption.

### Actigraphy and chronotype assessment

Habitual sleep–wake behaviour was assessed using wrist-worn actigraphy (Fitbit Inspire 3, Fitbit Inc., USA) over seven consecutive days prior to the first experimental trial, with monitoring maintained throughout the experimental period. Actigraphy data were used to derive sleep duration, sleep onset and wake time, sleep midpoint, and sleep efficiency (Haghayegh et al. 2019; Park et al. 2024; Stucky et al. 2021). Sleep midpoint was calculated as the midpoint between sleep onset and wake time and averaged across the monitoring period, and was used as a behavioural proxy of habitual circadian timing (Roenneberg et al. 2003, 2007; Wittmann et al. 2006). While a consumer-grade device was used, previous work has demonstrated acceptable agreement with research-grade actigraphy for sleep timing metrics, which were the primary variables of interest in the present study (Haghayegh et al. 2019; Park et al. 2024; Stucky et al. 2021). Participants also completed a validated Composite Morningness Questionnaire (CMQ) to determine circadian preference (Shahid et al. 2011). Actigraphy-derived sleep midpoint and questionnaire-based chronotype scores were included as exploratory variables to examine associations between circadian phenotype and time-of-day differences in performance.

### Familiarisation session

Participants attended a familiarisation session at least 48 h prior to the first experimental trial. Anthropometric measurements were recorded, including height (Seca 213 stadiometer, Seca GmbH & Co. KG., Germany), body mass (Seca GmbH & Co. KG., Hamburg, Germany), and cycle ergometer setup (including seat height and handlebar position), which was standardised and documented for replication in subsequent trials.

Participants completed a standardised warm-up consisting of 10 min of cycling at a self-selected moderate intensity (corresponding to a rating of perceived exertion of ~ 11–13 on the Borg 6–20 scale (Borg 1982), followed by a practice 10-minute cycling time trial to familiarise themselves with the pacing strategy and perceptual demands of the protocol, thereby minimising potential learning effects. Verbal instructions and feedback procedures were standardised during familiarisation and replicated across all experimental trials.

### Exercise protocol

All exercise trials were performed on a commercially available air-braked cycle ergometer (Wattbike Ltd., Nottingham, UK). Individual bike setup (seat height, handlebar position, and other adjustable components) was standardised for each individual during the familiarisation session and replicated across all experimental trials. Resistance settings (air brake level and magnetic resistance) were standardised for each participant during familiarisation and replicated across all subsequent trials. Environmental conditions (laboratory temperature 21 ± 2 °C, relative humidity 59 ± 6%) were recorded and maintained within a consistent range across visits.

Each session began with a standardised warm-up consisting of 10 min of cycling at a self-selected moderate intensity (RPE ~ 11–13), followed by a 2-minute passive recovery period. Participants then completed a 10-minute self-paced cycling time trial, during which they were instructed to produce the highest possible average power output over the duration of the trial. Standardised instructions were provided prior to each trial, emphasising that the goal was to maximise total work performed across the 10-minute period.

Visual feedback was restricted to elapsed time only, with no information provided regarding power output, cadence, or distance, to minimise pacing bias. The Wattbike performance monitor was configured accordingly for all trials. Standardised verbal encouragement was provided at regular intervals throughout the test. Power output (W), cadence (rpm), and distance (km) were recorded throughout the trial. Mean power output across the 10-minute trial was designated a priori as the primary performance outcome, with peak power output and total distance covered reported as secondary performance measures.

### Physiological and perceptual measures

Heart rate was recorded continuously throughout each trial using a chest-strap heart rate monitor (Polar H10, Polar Electro Oy, Kempele, Finland) and expressed as mean and peak values during the time trial. Capillary blood samples were obtained via fingertip puncture immediately before exercise and within 1 min following completion of the time trial. Blood lactate concentration was analysed using a portable lactate analyser (Lactate Pro 2, Arkray Inc., Kyoto, Japan). Rating of perceived exertion (RPE) was assessed immediately following completion of the time trial using the Borg 6–20 scale (Borg 1982). Body temperature was measured pre- and post-exercise using a tympanic thermometer (Braun ThermoScan IRT 4520, Braun GmbH, Germany), with measurements performed according to manufacturer guidelines under standardised conditions. All measurements were conducted in the same order and by the same investigator to minimise inter-observer variability. The timing of all measurements relative to exercise was standardised across trials.

### Statistical analysis

All statistical analyses were performed using GraphPad Prism (version 11.0.1; GraphPad Software, San Diego, CA, USA). Data were assessed for normality using the Shapiro–Wilk test prior to analysis. Descriptive data are presented as mean ± standard deviation (SD) unless otherwise stated. Statistical significance was set at *p* < 0.05.

The primary outcome, mean power output during the 10-minute time trial, was compared between morning (AM) and evening (PM) conditions using paired t-tests. To examine potential sex differences, a mixed-effects model was used with time of day (AM vs. PM) as a within-subject factor and sex (male vs. female) as a between-subject factor, with participant included as a random effect. Secondary performance outcomes, including peak power output and total distance covered, were analysed using paired t-tests.

Heart rate responses across the 10-minute time trial were analysed using a two-way repeated-measures analysis of variance (ANOVA). Heart rate data were averaged for each minute of the trial, resulting in 10 time points per participant. Time (1–10 min) and condition (AM vs. PM) were included as within-subject factors. Blood lactate, core temperature, and rating of perceived exertion (RPE) were analysed using two-way repeated-measures ANOVA, with time (pre vs. post) and condition (AM vs. PM) as within-subject factors. Where significant main effects or interactions were identified, post hoc comparisons with Šidák adjustment were performed. This approach was applied to all post hoc comparisons arising from ANOVA analyses, including chronotype-group comparisons presented in Fig. 2C. For blood lactate and core temperature, change scores (post-exercise minus pre-exercise) were calculated and used for subsequent analyses where appropriate.

Associations between circadian phenotype variables (chronotype score and actigraphy-derived sleep midpoint) and the diurnal difference in performance outcomes (PM minus AM) were assessed using simple linear regression. Pearson correlation coefficients (*r*) and coefficients of determination (R²) were reported to quantify the strength of these relationships. Potential trial order effects were explored, and exact *p* values, mean differences, and 95% confidence intervals were reported where appropriate.

## Results

### Exercise performance

**Fig. 1:**
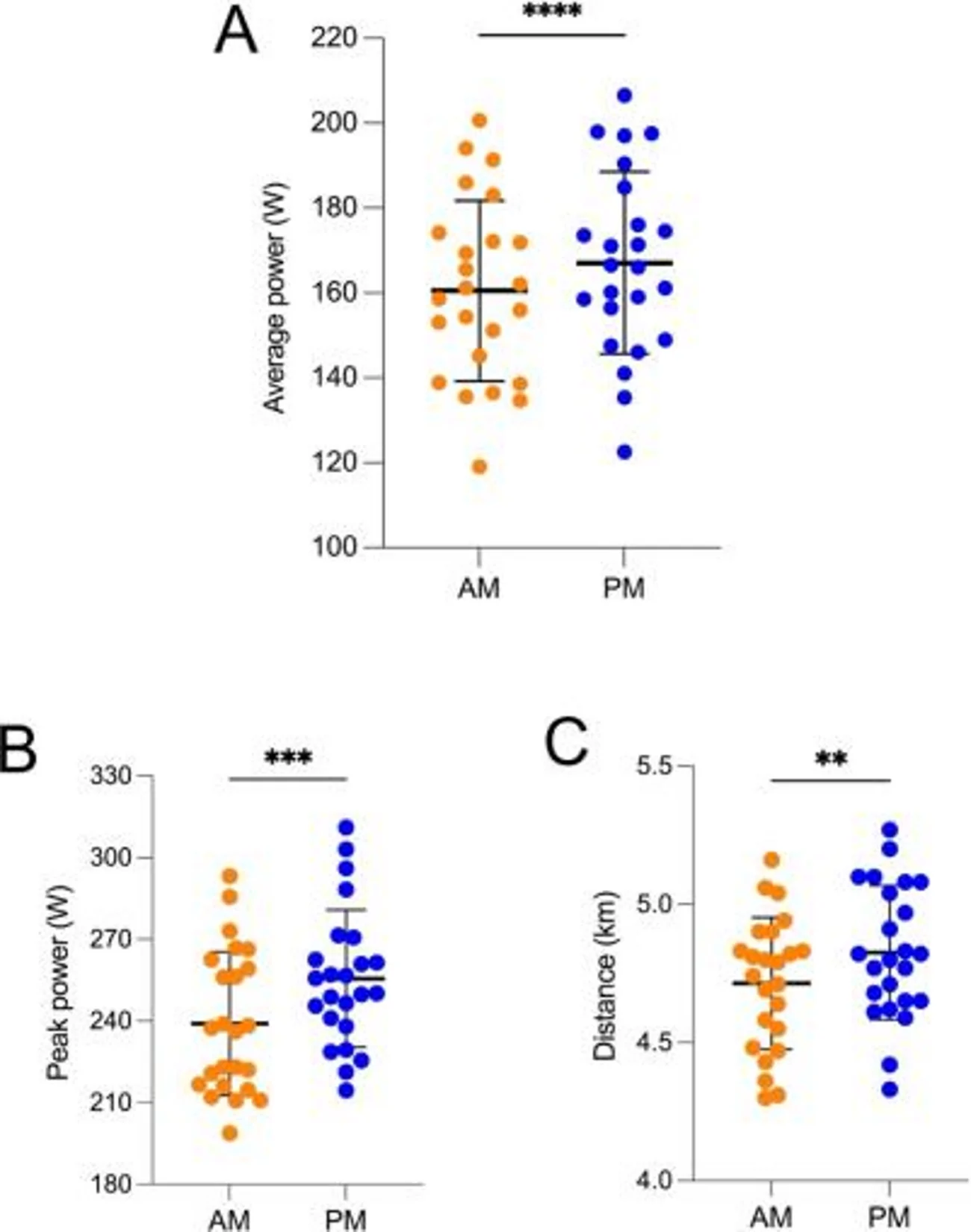
Diurnal variation in exercise performance during a 10-minute cycling time trial. Exercise performance outcomes measured during morning (AM) and evening (PM) trials in healthy young adults (*n* = 24). **(A)** Mean power output (W) achieved during the 10-minute time trial. **(B)** Peak power output (W) recorded during the time trial. **(C)** Total distance covered (km) during the time trial. Data are presented as individual responses with group mean ± SD overlaid. Each point represents a single participant, with paired observations shown for AM (orange) and PM (blue) conditions. Performance was significantly higher in the PM compared with the AM trial for all outcomes (paired t-tests): mean power (**** *p* < 0.0001), peak power (*** *p* = 0.0005), and distance covered (** *p* = 0.0049)

Mean power output during the 10-minute time trial was significantly higher in the evening (PM) compared with the morning (AM) (mean difference: 6.57 W, 95% CI: 4.99 to 8.15 W; *p* < 0.0001; Fig. 1A). Peak power output was also greater in the PM condition (mean difference: 16.48 W, 95% CI: 8.05 to 24.90 W; *p* = 0.0005; Fig. 1B). Similarly, total distance covered during the time trial was higher in the PM trial compared with AM (mean difference: 0.11 km, 95% CI: 0.04 to 0.18 km; *p* = 0.0049; Fig. 1C). Two-way analysis of variance confirmed a significant main effect of time-of-day (*p* < 0.0001) and sex (*p* < 0.0001) on mean power output, with no interaction between factors (*p* = 0.363), indicating that the diurnal performance effect was consistent across males and females.

Associations with circadian phenotype.

**Fig. 2:**
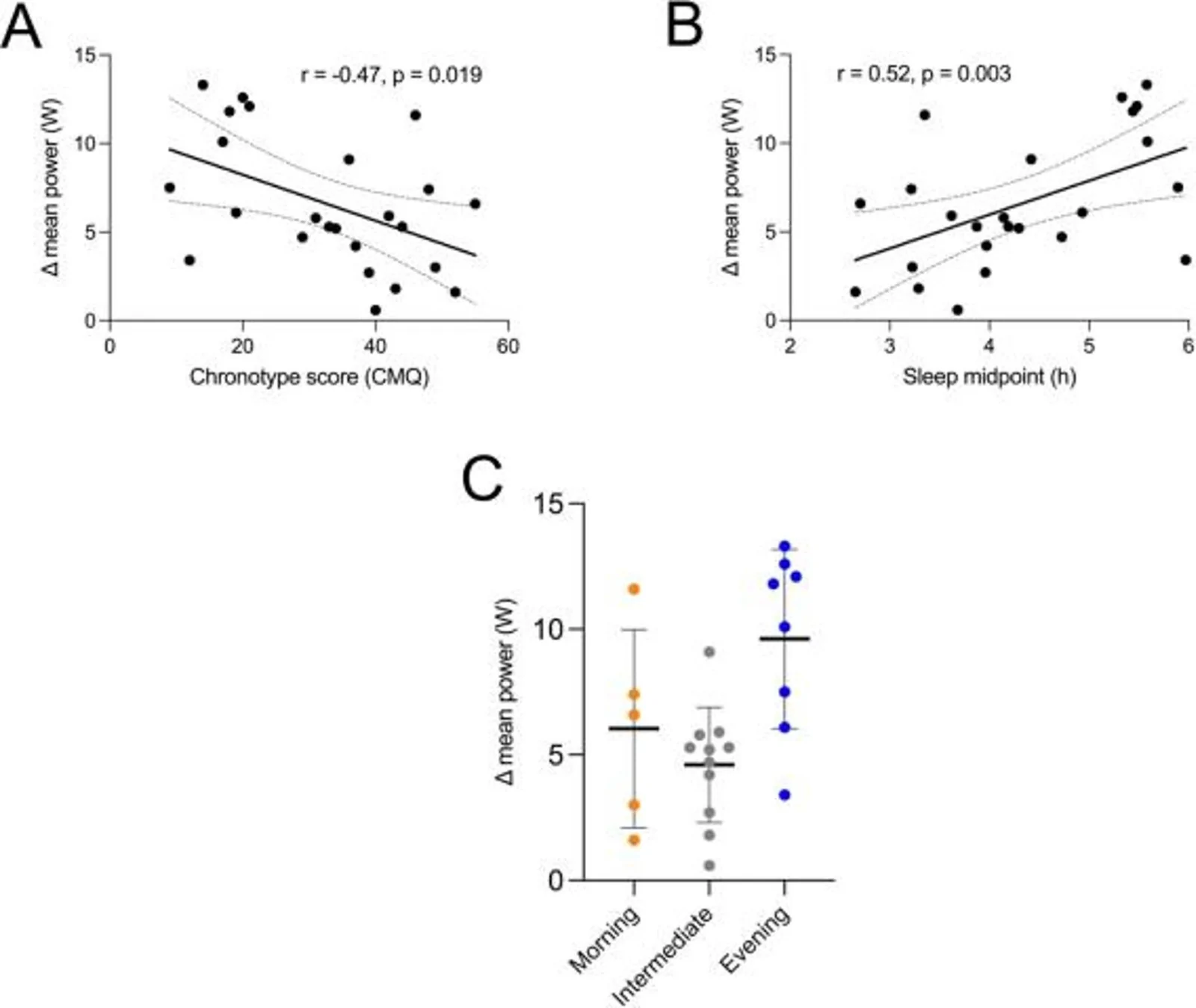
Associations between circadian phenotype and diurnal variation in exercise performance. Relationships between circadian phenotype and the diurnal difference in mean power output (PM − AM) during the 10-minute cycling time trial (*n* = 24). **(A)** Association between chronotype score (Composite Morningness Questionnaire, CMQ) and the diurnal difference in mean power output. A significant negative relationship was observed (*r* = − 0.47, *p* = 0.019), indicating that individuals with a more evening-oriented chronotype exhibited greater improvements in performance in the PM condition. **(B)** Association between actigraphy-derived sleep midpoint and the diurnal difference in mean power output. A significant positive relationship was observed (*r* = 0.52, *p* = 0.0093), indicating that later sleep timing was associated with a greater PM–AM performance difference. In panels A and B, solid lines represent linear regression fits, with dashed lines indicating 95% confidence intervals. Each point represents an individual participant. **(C)** Diurnal difference in mean power output stratified by chronotype category (morning, intermediate, evening). Data are presented as individual responses with group mean ± SD overlaid. A significant main effect of chronotype was observed (*p* = 0.0082), with a trend towards greater diurnal variation in evening-type individuals

There was a significant negative association between chronotype score (CMQ) and the diurnal difference in mean power output (PM–AM) (*r* = − 0.47, *p* = 0.019; Fig. 2A), indicating that individuals with a more evening-oriented chronotype demonstrated greater improvements in performance in the PM condition. Chronotype accounted for 22% of the variance in the magnitude of diurnal performance differences (R² = 0.224). Actigraphy-derived sleep midpoint was also significantly associated with the diurnal difference in performance (*r* = 0.52, *p* = 0.0093; Fig. 2B), with later sleep timing associated with greater PM–AM performance differences. Sleep midpoint accounted for 27% of the variance in diurnal performance responses (R² = 0.270). When participants were grouped according to chronotype category, there was a significant effect of chronotype on the magnitude of diurnal variation in performance (*p* = 0.0082; Fig. 2C). Although post hoc comparisons did not reach statistical significance following correction (morning vs. evening: *p* = 0.057), there was a clear trend towards greater PM–AM performance differences in individuals with an evening chronotype.

Physiological responses.

**Fig. 3:**
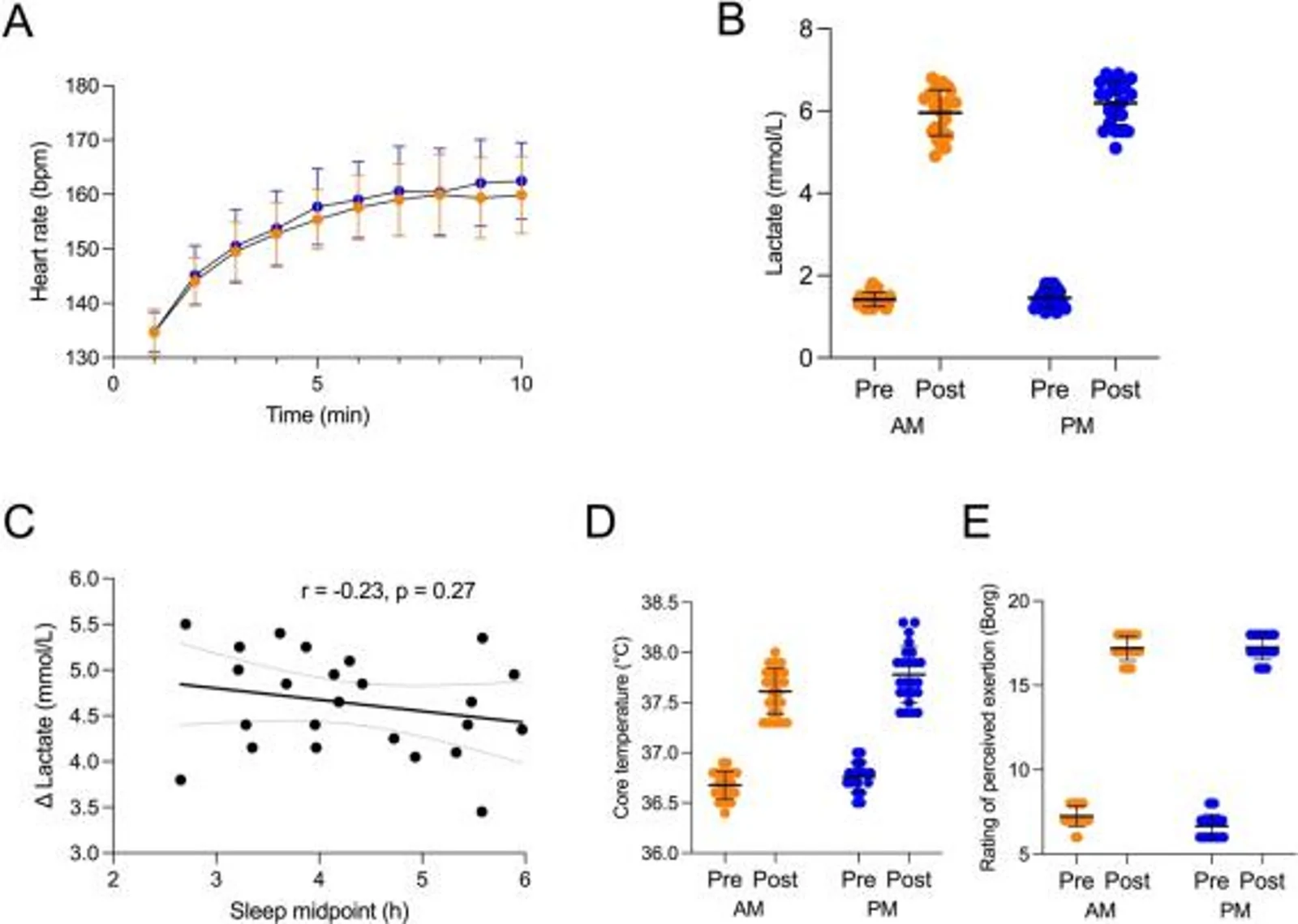
Physiological responses to exercise during morning and evening trials. Physiological responses measured during the 10-minute cycling time trial in morning (AM) and evening (PM) conditions (*n* = 24). **(A)** Heart rate (bpm) recorded throughout the 10-minute time trial. Values are presented as mean ± SD at each time point. Heart rate increased over time (*p* < 0.0001), with no effect of time of day (*p* = 0.401) and no interaction between time and condition (*p* = 0.495). **(B)** Blood lactate concentration (mmol·L⁻¹) measured pre- and post-exercise in AM and PM trials. Lactate increased significantly following exercise (*p* < 0.0001), with no difference between conditions (*p* = 0.112) and no interaction (*p* = 0.263). **(C)** Association between sleep midpoint and the exercise-induced change in blood lactate (Δ lactate; post − pre). No significant relationship was observed (*r* = − 0.23, *p* = 0.27). Solid line represents linear regression, with dashed lines indicating 95% confidence intervals. **(D)** Core temperature (°C) measured pre- and post-exercise in AM and PM trials. Core temperature increased significantly following exercise (*p* < 0.0001), with a small but significant effect of time of day (*p* = 0.026) and no interaction (*p* = 0.104). **(E)** Rating of perceived exertion (RPE; Borg scale) measured pre- and post-exercise. RPE increased significantly following exercise (*p* < 0.0001), with no main effect of time of day (*p* = 0.061) but a small interaction effect (*p* = 0.033). In panels B, D, and E, data are presented as individual responses with group mean ± SD overlaid. Each point represents a single participant, with paired observations shown for AM (orange) and PM (blue) conditions

Heart rate increased progressively during the time trial (*p* < 0.0001), with no effect of time of day (*p* = 0.401) and no interaction between time and condition (*p* = 0.495; Fig. 3A). Blood lactate concentration increased markedly from pre- to post-exercise (*p* < 0.0001), with no difference between AM and PM conditions (*p* = 0.112) and no interaction (*p* = 0.263; Fig. 3B). To explore whether inter-individual variability in metabolic responses to exercise might contribute to the observed relationship between circadian phenotype and performance, the exercise-induced change in blood lactate concentration (Δ lactate; post-exercise minus pre-exercise, averaged across AM and PM trials) was examined in relation to sleep midpoint. No significant association was observed (*r* = − 0.23, *p* = 0.27), only accounting for approximately 5% of the variance (R² = 0.055; Fig. 3C). Core temperature increased significantly following exercise (*p* < 0.0001), with a small but statistically significant effect of time-of-day (*p* = 0.026), reflecting higher temperatures in the PM condition. There was no interaction between time and condition (*p* = 0.104; Fig. 3D). Rating of perceived exertion increased substantially from pre- to post-exercise (*p* < 0.0001). There was no main effect of time-of-day (*p* = 0.061), although a small but statistically significant interaction was observed (*p* = 0.033), indicating a modest difference in the magnitude of perceptual response between AM and PM trials (Fig. 3E).

## Discussion

The present study demonstrates that short-duration endurance performance was enhanced in the evening compared with the morning in healthy young adults, with consistent increases in mean power, peak power, and total distance covered (Fig. 1). Importantly, these diurnal differences occurred despite comparable heart rate and blood lactate responses between conditions (Fig. 3), suggesting that the observed performance effect was not accompanied by substantial differences in systemic cardiovascular or metabolic strain. Exploratory analyses further demonstrate that both chronotype (CMQ) and actigraphy-derived sleep timing were significantly associated with the magnitude of the PM-AM performance difference, accounting for ~ 22–27% of the variance in the observed response (Fig. 2). Collectively, these findings support the presence of a robust time-of-day effect on exercise performance and extend previous work linking circadian phenotype to diurnal variation in physical performance (Chtourou et al. 2012; Facer-Childs and Brandstaetter 2015; J. A. Vitale et al. 2017). However, because each participant completed only a single morning and evening trial, the observed associations should be interpreted cautiously and not as evidence of stable individual responses.

The observation of superior evening performance is consistent with a substantial body of work demonstrating diurnal variation in physical performance, typically characterised by peak outputs in the late afternoon or early evening (Hesketh and Esser 2024). Early work by Hill et al. (1989) and (Reilly et al. 2007 identified time-of-day effects across a range of performance tasks, while more recent controlled studies have reinforced these findings in both aerobic and anaerobic modalities (Chtourou et al. 2012). In particular, Chtourou et al. (2012) reported greater peak and mean power output during repeated sprint exercise in the evening, alongside elevated muscle temperature, suggesting a physiological basis for enhanced performance capacity later in the day. Similarly, Souissi et al. (2004, 2007) demonstrated improved Wingate performance in the evening, with diurnal differences closely aligned to fluctuations in core temperature. The present data align with these observations, demonstrating consistent improvements in endurance-based performance in the evening, with mean power output ~ 6–8% higher in the PM compared with the AM condition (Fig. 1A–C).

In contrast to previous work (Rae et al. 2015), the current findings suggest that these performance differences are not explained by large alterations in systemic cardiovascular or metabolic strain (Drust et al. 2005; Rae et al. 2015). Despite a clear rise in core temperature from pre- to post-exercise and slightly higher absolute values in the PM condition (Fig. 3D), neither heart rate nor lactate responses differed between AM and PM trials (Fig. 3). This dissociation between performance output and commonly measured physiological markers suggests that diurnal variation in performance may not be fully captured by whole-body indices of strain, and may instead reflect more complex integrative processes. This interpretation is consistent with findings by Rae et al. (2015), who reported time-of-day differences in maximal strength independent of neuromuscular activation, suggesting that central or neuromuscular factors may contribute to diurnal performance variation.

From a mechanistic perspective, these findings are also consistent with emerging work highlighting the importance of circadian timing in the regulation of exercise metabolism and adaptation (Ezagouri et al. 2019; Sato et al. 2019). Ezagouri et al. (2019) demonstrated that exercise capacity and metabolic responses vary according to circadian phase in both animal models and humans, with distinct molecular signatures observed depending on the timing of exercise. While the present study did not assess molecular outcomes, the absence of large differences in systemic physiological markers alongside clear performance differences may reflect underlying circadian regulation of skeletal muscle function or substrate utilisation that is not captured by the measures employed here. However, such interpretations remain inferential and require direct investigation.

Emerging evidence from rodent models suggests that both central and peripheral circadian clocks may contribute to time-of-day variation in exercise performance (Ezagouri et al. 2019; Zhang et al. 2026). While the suprachiasmatic nucleus coordinates systemic circadian rhythms and influences behavioural factors such as arousal, sleep–wake timing, and motivation (Takahashi 2017), skeletal muscle possesses an autonomous molecular clock that regulates key aspects of muscle physiology including substrate metabolism, mitochondrial function, excitation–contraction coupling, and contractile performance (Dyar et al. 2018; Zhang et al. 2026). Studies using tissue-specific clock disruption models have demonstrated that impairment of skeletal muscle clock function can alter exercise capacity and metabolic responses, supporting a direct role for peripheral circadian regulation of muscle performance (Andrews et al. 2010; Hodge et al. 2015). Although the present study cannot distinguish between central and peripheral clock contributions, the observed association between behavioural circadian phenotype and performance variability is consistent with the concept that interactions between central circadian timing and local skeletal muscle clock mechanisms may contribute to diurnal variation in human exercise performance.

A key strength of the present study is the tightly controlled, within-subject human design, incorporating standardisation of sleep, diet, and prior activity, alongside objective assessment of circadian behaviour using actigraphy. This approach reduces many of the confounding factors that have limited interpretation in earlier studies of diurnal performance, where variability in sleep timing, habitual activity, or prior exertion may have contributed to observed effects. The use of a randomised, counterbalanced crossover design further strengthens the internal validity of the findings and allows for more confident attribution of observed differences to time-of-day effects.

A particularly important aspect of the current work is the integration of circadian phenotype into the analysis of performance variability (Kang et al. 2023). While diurnal variation in performance is well established, relatively few studies have explicitly examined the role of chronotype or behavioural circadian markers in modulating these responses. The present findings demonstrate that both self-reported chronotype and actigraphy-derived sleep timing are significantly associated with the magnitude of diurnal performance variation (Fig. 2), accounting for a meaningful proportion of the variance in the observed PM-AM performance difference. These observations are consistent with previous work demonstrating that circadian phenotype influences both physical and cognitive performance across the day (Facer-Childs and Brandstaetter 2015; J. A. Vitale et al. 2017).

Importantly, the current data extend these findings by quantifying the extent to which behavioural proxies of circadian timing are associated with performance variability under controlled experimental conditions. The association between sleep midpoint and performance (Fig. 2B) suggests that habitual sleep–wake timing may serve as a practical proxy for circadian alignment in applied settings. However, it should be acknowledged that these measures reflect behavioural, rather than endogenous, circadian phase, and therefore do not allow direct inference regarding underlying circadian biology. The absence of a relationship between circadian phenotype and physiological responses such as lactate (Fig. 3C) further suggests that diurnal variation in performance may not be driven solely by peripheral metabolic factors, but may instead reflect interactions between behavioural timing, central regulation, and task-specific demands. Furthermore, the absence of a relationship between circadian phenotype and exercise-induced lactate responses suggests that the observed performance differences are unlikely to be explained by simple differences in whole-body metabolic strain, supporting the possibility that other physiological or circadian mechanism contribute to inter-individual variability in time-of-day performance responses.

While behavioural measures such as chronotype questionnaires and actigraphy-derived sleep timing provide practical and ecologically relevant indicators of circadian phenotype, they do not directly assess endogenous circadian phase or molecular clock function. Physiological markers including melatonin and cortisol rhythms offer complementary information regarding internal circadian timing and may provide additional insight into the mechanisms underlying time-of-day variation in exercise performance. Similarly, assessment of molecular clock gene expression within relevant tissues such as skeletal muscle may help establish whether behavioural circadian phenotype reflects differences in local circadian organisation that influence muscle function and metabolism. Future studies should therefore seek to integrate behavioural, endocrine, and molecular measures of circadian timing to more comprehensively characterise the relationship between circadian biology and exercise performance and to better understand the sources of inter-individual variability in diurnal exercise responses.

From an applied perspective, our findings have implications for the optimisation of exercise timing (Guest et al. 2021). While the magnitude of the performance difference observed is modest, it is consistent and systematic, and comparable to changes reported following commonly used acute interventions in exercise settings (Guest et al. 2021; López-González et al. 2018; Singh and Hesketh 2026). As such, these differences may be meaningful in both competitive and clinical contexts. Furthermore, the observed variation in response highlights the potential importance of personalised approaches to exercise prescription, taking into account circadian phenotype and habitual sleep behaviour. This aligns with the emerging concept of “chrono-exercise medicine,” in which exercise timing is considered as a modifiable factor influencing performance and health outcomes.

Several limitations should be acknowledged. The study was conducted in a relatively small sample of young, healthy adults, and the extent to which these findings generalise to older individuals, clinical populations, or highly trained athletes remains to be determined. In addition, while the study was adequately powered to detect time-of-day differences in performance, analyses relating to circadian phenotype should be considered exploratory, particularly as each participant completed only a single morning and evening trial. Consequently, the observed associations may partly reflect day-to-day biological or behavioural variability in addition to stable individual differences. Although sleep timing was assessed using actigraphy, more precise determination of circadian phase (e.g., dim light melatonin onset) was not performed. Finally, while sex was included as a factor in the analysis, the study was not specifically powered to detect sex-specific differences in diurnal responses.

In conclusion, the present study confirms that endurance exercise performance is enhanced in the evening compared with the morning and demonstrates that the magnitude of this effect is associated with measures of circadian phenotype. These findings highlight the importance of considering both time-of-day and circadian alignment in the assessment of human performance and support the growing view that exercise timing represents a relevant dimension of personalised exercise prescription. Future work should seek to integrate molecular, physiological, and behavioural measures to further elucidate the mechanisms underpinning these effects and to determine how exercise timing can be optimised to improve both performance and health outcomes.

## Data Availability

The data supporting the findings of this study are available in the Figshare repository, DOI: https://doi.org/10.6084/m9.figshare.32129587.

## Abbreviations

AM: Morning
ANOVA: Analysis of variance
CMQ: Composite Morningness Questionnaire
HR: Heart rate
PM: Evening
RPE: Rating of perceived exertion
TT: Time trial
