# Correction: Circadian phenotype is associated with diurnal variation in human exercise performance

**DOI:** 10.1007/s00421-026-06393-5

**Published:** 2026-09-01

**Authors:** Akshay Singh, Sanjida Ahmed, Stuart J. Hesketh

**Affiliations:** School of Medicine and Dentistry, University of Lancashire, Victoria Street, Harrington Building, , Preston, PR1 2HE UK

**Correction: European Journal of Applied Physiology**

**https://doi.org/10.1007/s00421-026-06352-0**

In the original version of this article, all figure legends were incorporated into the main body of text rather than placing underneath the figure caption. The Figs. 1, 2 and 3 caption should have read

Fig. 1. Diurnal variation in exercise performance during a 10-min cycling time trial. Exercise performance outcomes measured during morning (AM) and evening (PM) trials in healthy young adults (*n* = 24). (**A**) Mean power output (W) achieved during the 10-min time trial. (**B**) Peak power output (W) recorded during the time trial. (**C**) Total distance covered (km) during the time trial. Data are presented as individual responses with group mean ± SD overlaid. Each point represents a single participant, with paired observations shown for AM (orange) and PM (blue) conditions. Performance was significantly higher in the PM compared with the AM trial for all outcomes (paired *t*-tests): mean power (****p < 0.0001), peak power (***p = 0.0005), and distance covered (**p = 0.0049).

Fig. 2. Associations between circadian phenotype and diurnal variation in exercise performance. Relationships between circadian phenotype and the diurnal difference in mean power output (PM–AM) during the 10-min cycling time trial (*n* = 24). (**A**) Association between chronotype score (Composite Morningness Questionnaire, CMQ) and the diurnal difference in mean power output. A significant negative relationship was observed (*r* = − 0.47, *p* = 0.019), indicating that individuals with a more evening-oriented chronotype exhibited greater improvements in performance in the PM condition. (**B**) Association between actigraphy-derived sleep midpoint and the diurnal difference in mean power output. A significant positive relationship was observed (*r* = 0.52, *p* = 0.0093), indicating that later sleep timing was associated with a greater PM–AM performance difference. In panels **A** and **B**, solid lines represent linear regression fits, with dashed lines indicating 95% confidence intervals. Each point represents an individual participant. (**C**) Diurnal difference in mean power output stratified by chronotype category (morning, intermediate, evening). Data are presented as individual responses with group mean ± SD overlaid. A significant main effect of chronotype was observed (*p* = 0.0082), with a trend towards greater diurnal variation in evening-type individuals.

Fig. 3. Physiological responses to exercise during morning and evening trials. Physiological responses measured during the 10-min cycling time trial in morning (AM) and evening (PM) conditions (*n* = 24). (**A**) Heart rate (bpm) recorded throughout the 10-min time trial. Values are presented as mean ± SD at each time point. Heart rate increased over time (*p* < 0.0001), with no effect of time of day (*p* = 0.401) and no interaction between time and condition (*p* = 0.495). (**B**) Blood lactate concentration (mmol·L⁻¹) measured pre- and post-exercise in AM and PM trials. Lactate increased significantly following exercise (*p* < 0.0001), with no difference between conditions (*p* = 0.112) and no interaction (*p* = 0.263). (**C**) Association between sleep midpoint and the exercise-induced change in blood lactate (Δ lactate; post–pre). No significant relationship was observed (*r* = − 0.23, *p* = 0.27). Solid line represents linear regression, with dashed lines indicating 95% confidence intervals. (**D**) Core temperature (°C) measured pre- and post-exercise in AM and PM trials. Core temperature increased significantly following exercise (*p* < 0.0001), with a small but significant effect of time of day (*p* = 0.026) and no interaction (*p* = 0.104). (**E**) Rating of perceived exertion (RPE; Borg scale) measured pre- and post-exercise. RPE increased significantly following exercise (*p* < 0.0001), with no main effect of time of day (*p* = 0.061) but a small interaction effect (*p* = 0.033). In panels **B**, **D**, and **E**, data are presented as individual responses with group mean ± SD overlaid. Each point represents a single participant, with paired observations shown for AM (orange) and PM (blue) conditions.

The original article has been corrected.

